# Applications of Artificial Intelligence–Driven Chatbots in University Level Dental Education: A Systematic Review

**DOI:** 10.1155/ijod/6612348

**Published:** 2026-11-01

**Authors:** Ramnarayan B. K., Raghad Hashim, Krishnanand P. S., Vidya M. A., Alexander Maniangat Luke, Usha C.

**Affiliations:** ^1^ Department of Oral Medicine and Radiology Dayananda Sagar College of Dental Sciences Kumaraswamy Layout Bangalore 560111 India dayanandasagar.edu; ^2^ Department of Basic Medical and Dental Sciences College of Dentistry, Centre of Medical and Bio-Allied Health Science Research Ajman University Al Jurf, P.O. Box 346 Ajman UAE ajman.ac.ae; ^3^ Department of Oral Pathology Dayananda Sagar College of Dental Sciences Kumaraswamy Layout Bangalore 560111 India dayanandasagar.edu; ^4^ Department of Clinical Science College of Dentistry, Centre of Medical and Bio-Allied Health Science Research Al Jurf, P.O. Box 346 Ajman UAE; ^5^ Oral Medicine and Radiology Department of Dental Surgery CAPFIMS AIIMS New Delhi India aiims.edu

**Keywords:** artificial intelligence, chatbots, dental education, large language models, simulation, systematic review, virtual patients

## Abstract

**Background:**

Artificial intelligence (AI), mainly AI‐driven chatbots and large language models (LLMs) is reshaping dental education through adaptive, interactive, and simulation‐based learning. While the transition in technology has been rapid, the evidence about the educational effectiveness of these technologies is still very diverse and not entirely directed.

**Aim:**

To analyze the roles, educational effectiveness, and impact of integrating AI‐driven chatbots and LLMs in dental education at the university level with a focus on learning outcomes and implementation practices.

**Methods:**

The researchers conducted a thorough search of the electronic databases PubMed/MEDLINE, Scopus, Web of Science, and Embase for articles published from January 2018 to November 2025. This review met the Preferred Reporting Items for Systematic Reviews and Meta‐Analyses (PRISMA) 2020 criteria and was made up of original research articles on dental studentsand educators who were using AI‐based chatbots as interventions. The outcomes measured were knowledge gain, clinical reasoning, diagnostic accuracy, communication skills, and engagement of learners. Due to the differences in the methodologies used the authors opted for a narrative synthesis approach.

**Results:**

Six studies met the eligibility criteria and showed potential benefits of AI‐driven chatbots and LLMs for diagnostic reasoning, learning performance, engagement, and individualized feedback although methodological and outcome heterogeneity limited direct comparison.

**Conclusion:**

AI‐driven chatbots and LLMs may complement university‐level dental education, but their effectiveness and safe implementation require further rigorous evaluation.

## 1. Introduction

Artificial intelligence (AI) is broadly recognized as a transformative technology crossing many healthcare domains, and dentistry is one of the fields where we can observe the quick appearance of new useful applications for clinical practice, decision support, and education [1].

Consequently, AI‐based tools, in particular chatbots and large language models (LLMs) (advanced deep learning models like GPT‐based systems) have entered the dental profession not only as a means of helping diagnostic reasoning, treatment planning, and patient communication but also with a set of purposes including achieving higher safety, faster, and increased access to care [2].

The fact that clinical utility of AI is becoming more evident does not mean that the implications of these technologies for academic training and critical thinking development in dental education are not worth further research [2, 3].

Chatbots like ChatGPT have been found to be helpful tools in increasing student involvement, giving students quick access to information, and even helping students learn by making patient simulations. Nevertheless, there still remain certain questions about accuracy, dependability, and moral use, especially when AI‐produced material is used for academic evaluations or clinical decision making [4–6]. Some recent research have acknowledged the potential as well as the drawbacks of AI systems in dentistry and the whole healthcare area and have called for a thorough examination of their performance, educational impact, and integration methods [6, 7]. Besides that, there has been such a rapid growth of generative AI technologies without the existence of any standard guidelines, which has led to important issues being raised about curriculum alignment, development of critical thinking, and the responsible use of these technologies in dental training settings [3, 8].

While there has been growing focus on AI in the dental field, the literature about the effectiveness of AI as a learning tool, notably in higher education dental courses, is still scattered and diverse. The difference in study designs, the results of learning, and the way of assessment among the existing studies have been so great that it is impossible to generalize the findings and base the integration of the curricula on the evidence [1–8].

This systematic review aimed to evaluate the educational functions, effectiveness, and implementation challenges of AI‐driven chatbots and LLMs in university‐level dental education, with an emphasis on learning outcomes and practical integration into teaching and training.

## 2. Materials and Methods

### 2.1. Study Design and Reporting Framework

The systematic review was conducted per the next guidelines: Preferred Reporting Items for Systematic Reviews and Meta‐Analyses (PRISMA 2020).The protocol was registered with Open Science Framework (OSF) with DOI 10.17605/OSF.IO/8AEVF. The review was pre‐planned, and the protocol was documented to ensure transparency, reproducibility, and good methodological practices.

### 2.2. Research Question

What are the educational functions, effectiveness, and implementation challenges of AI‐driven chatbots and LLMs in university‐level dental education?

#### 2.2.1. Eligibility Criteria

Inclusion and exclusion criteria were determined prior to study, and they were based on the PICO model to ensure that they were aligned with the review aim. Only empirical papers that assessed the use of AI‐based chatbots or LLMs as education tools in dental schools were considered. The eligible studies might have involved dental students at the bachelor or master levels, dental interns, or dental teachers, and they should have presented at least one quantifiable educational outcome, for example, knowledge gain, clinical reasoning, diagnostic accuracy, communication skills, or learning performance.

Excluded studies included those focusing on non‐educational AI applications, conducted outside dental educational settings, lacking measurable educational outcomes, or reported as nonempirical publications (reviews, editorials, commentaries, or preprints).

Only studies relevant to educational effectiveness and the review question were included; studies were categorized as chatbot‐based or LLM‐based interventions, and no subgroup analysis was conducted due to the limited number of included studies.

The PICO framework was used to structure the research question (Table 1).

**Table 1:** PICO question for AI chatbots in dental education.

PICO element	Description
Population (P)	Undergraduate and postgraduate dental students, dental interns, and dental educators involved in university‐level dental education
Intervention (I)	Use of artificial intelligence (AI)–based chatbots or large language models (LLMs) as educational interventions (e.g., virtual patient simulations, adaptive tutoring systems, communication training tools)
Comparison (C)	Traditional teaching methods, standard curriculum, no AI intervention, or other digital learning tools
Outcomes (O)	Primary outcomes: knowledge acquisition, clinical reasoning, diagnostic accuracy, communication skills, and learning performance metrics (quantifiable). Secondary outcomes: learner engagement, satisfaction, feasibility, and acceptability

### 2.3. Information Sources

A comprehensive electronic search was conducted across four major databases:

• PubMed/MEDLINE
• Scopus
• Web of Science Core Collection
• Embase

The final search was conducted on 30 November 2025. In addition, reference lists of included studies were manually screened to identify potentially eligible articles.

### 2.4. Search Strategy

The authors combined controlled vocabulary terms (MeSH and Emtree) with free‐text keywords related to AI chatbots LLMs and dental education in their search strategy. The search was conducted for publications between January 2018 and November 2025 and was limited to English‐language studies (Table 2).

**Table 2:** Detailed search strategy across databases.

Database	Search date	Search strategy (boolean string)
PubMed/MEDLINE	30 November 2025	(“Artificial Intelligence”[Mesh] OR “artificial intelligence” OR “AI” OR “chatbot ^∗^” OR “large language model ^∗^” OR “LLM” OR “ChatGPT”) AND (“Education, Dental”[Mesh] OR “dental education” OR “dental student ^∗^” OR “dental curriculum” OR “dental training”) AND (“learning” OR “teaching” OR “simulation” OR “virtual patient” OR “clinical reasoning”) Filters: English; 2018–2025
Scopus	30 November 2025	TITLE‐ABS‐KEY (“artificial intelligence” OR AI OR chatbot ^∗^ OR “large language model ^∗^” OR LLM OR ChatGPT) AND TITLE‐ABS‐KEY (“dental education” OR “dental student ^∗^” OR “dental curriculum” OR “dental training”) AND TITLE‐ABS‐KEY (learning OR teaching OR simulation OR “virtual patient” OR “clinical reasoning”) AND PUBYEAR > 2017 AND (LIMIT‐TO (LANGUAGE, “English”))
Web of Science (Core Collection)	30 November 2025	TS = (“artificial intelligence” OR AI OR chatbot ^∗^ OR “large language model ^∗^” OR LLM OR ChatGPT) AND TS = (“dental education” OR “dental student ^∗^” OR “dental curriculum” OR “dental training”) AND TS = (learning OR teaching OR simulation OR “virtual patient” OR “clinical reasoning”) Refined by: English; 2018–2025
Embase	30 November 2025	(“artificial intelligence”/exp OR “artificial intelligence” OR AI OR chatbot ^∗^ OR “large language model ^∗^” OR LLM OR ChatGPT) AND (“dental education”/exp OR “dental student”/exp OR “dental curriculum” OR “dental training”) AND (learning OR teaching OR simulation OR “virtual patient” OR “clinical reasoning”) AND [english]/lim AND [2018–2025]/py

### 2.5. Study Selection Process

All retrieved records were exported into Rayyan software for duplicate removal and screening. Two independent reviewers performed the screening. Disagreements were resolved by discussion. Inter‐reviewer agreement was substantial (Cohen’s kappa = 0.82). The selection process is illustrated using a PRISMA 2020 flow diagram (Figure 1). Simulation‐based chatbot evaluations and AI‐generated assessment tools were categorized as in vitro studies, whereas studies involving real participants (students, educators, or patients) were classified as in vivo.

**Figure 1:**
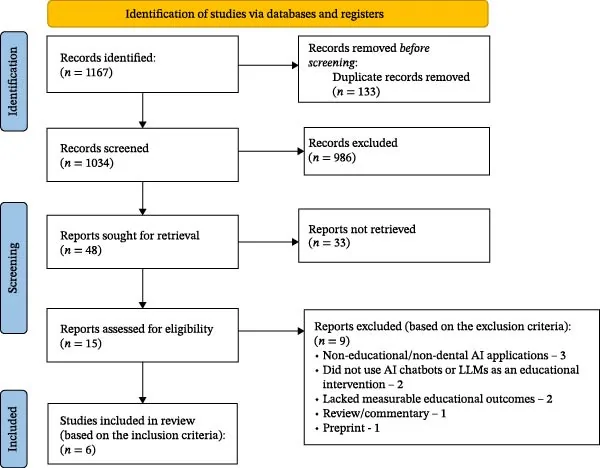
PRISMA 2020 flow diagram.

### 2.6. Data Extraction

A standardized data extraction form was developed. Extraction was conducted independently by two reviewers. The following variables were extracted:

• Author and year
• Country
• Study design
• Sample size
• Participant characteristics
• Type of AI intervention
• Comparator
• Educational domain
• Outcome measures
• Key findings
• Reported limitations.

### 2.7. Risk of Bias Assessment

The methodological quality of the included studies was assessed using design‐specific risk‐of‐bias tools to ensure appropriate evaluation across heterogeneous study designs. Randomized controlled trials were evaluated using the Cochrane Risk of Bias tool (RoB 2), while non‐randomized and comparative studies were assessed using the ROBINS‐I framework. Cross‐sectional studies were appraised using a structured assessment focusing on the sampling strategy, measurement validity, and reporting transparency. Qualitative and design‐based studies were evaluated based on the clarity of methodology, data credibility, and completeness of reporting.

Given the variability in reporting standards and study designs, a simplified traffic light system (low risk, some concerns, and high risk) was applied to enable consistent comparison across studies. Two independent reviewers conducted the risk‐of‐bias assessment, and any disagreements were resolved through discussion and consensus. The domains assessed included selection bias, performance bias (or confounding), outcome measurement bias, and reporting bias.

### 2.8. Data Synthesis

Studies were assessed based on how well the results could be generalized and if there were other factors that might have affected the results but were not considered in the study. A single‐method systematic review of this nature allowed for a thorough and transparent investigation of the problems related to a particular method without limiting oneself to overlooking the long‐standing weaknesses of various educational studies or those combining different types.

The studies were divided first into broad categories that correspond to their educational use: (1) virtual patient simulations, (2) adaptive tutoring and formative feedback systems, (3) communication and role‐play training, and (4) academic and research support tools. Then, within each category, we had a descriptive synthesis of the findings with a focus on the main features such as knowledge acquisition, diagnostic reasoning, communication skills, learner engagement, satisfaction, and feasibility.

There were differences in study designs, sample sizes, intervention characteristics, and measurement tools, which made it impossible to conduct a quantitative meta‐analysis. Therefore, the results were described narratively, focusing on patterns, consistencies, and variations among the studies. The six included studies evaluated AI‐derived chatbots or AI‐integrated educational applications across simulation‐based learning, classroom implementation, clinical learning, operative skill education, radiographic diagnostic training, and personalized feedback.

## 3. Results

Six studies met the predefined eligibility criteria and were included in the qualitative synthesis. The included evidence comprised experimental, implementation/comparative, mixed‐method, randomized controlled, randomized clinical, and controlled study designs. The studies evaluated AI‐driven chatbots and LLMs for virtual patient learning, clinical learning, diagnostic reasoning, instructor‐supported skills education, radiographic interpretation, and personalized feedback (Table 3).

**Table 3:** PICO evidence summary of included studies (AI chatbots in dental education).

Ref.	Author (year)/study category	Participants	Sample size	Intervention	Comparator	Outcome measure	Direction of effect	Key limitations
[9]	Suárez et al. (2022)—Simulation‐based student educational study	4th‐ and 5th‐year dental students at Universidad Europea de Madrid	193	AI‐powered chatbot simulating a virtual patient; students interacted with the virtual patient over several weeks and subsequently evaluated the experience	No formal comparator; descriptive educational evaluation	Student satisfaction, perceived usefulness, interaction quality, realism of communication, perceived improvement in communication/confidence, and overall acceptance using a 5‐point Likert questionnaire	Favorable: overall satisfaction with the interaction was high (mean 4.36/5); 5th‐year students reported higher satisfaction, and students who reached a correct diagnosis rated the technology more positively	Cross‐sectional/descriptive design; no control group; outcomes were predominantly self‐reported; single institution; short‐term perception rather than objective long‐term learning or clinical performance
[10]	Jones et al. (2025)—Classroom‐based implementation/usability study	Second‐ and final‐year students in a Bachelor of Oral Health programme	70 exposed (46 second‐year + 24 final‐year); 14 completed the usability questionnaire	GPT‐powered AI role‐play comprising interconnected virtual‐patient and clinical‐supervisor chatbots; implemented in classroom/seminar sessions	No formal control group; design‐based implementation study	Educator observations, student engagement, peer discussion, usability, authenticity/relevance, interface usability, perceived usefulness, and feasibility/cost	Generally favorable: AI role‐play promoted peer discussion and engagement; among survey completers, 86% strongly agreed it aligned with real‐world dental scenarios; however, preference over conventional teaching was mixed	Early design‐based research cycle; convenience sampling; small usability sample (*n* = 14); descriptive analysis; no objective comparison of learning outcomes; educator observations may be subjective; chatbot sometimes supplied excessive information or lacked authentic responsiveness
[11]	Fang et al. (2024)—Clinical learning/implementation and student‐experience study	Predoctoral dental students at University of Illinois Chicago College of Dentistry	86; chatbot *n* = 43, Blackboard *n* = 43	Custom‐developed chatbot used for clinical inquiries in a predoctoral implant clinic; 10–15‐min interaction based on 35 typical clinical questions	Traditional Blackboard (BB) online platform	AI awareness/perception, timeliness, interaction, receptiveness, anxiety, satisfaction, perceived faculty workload, and concerns regarding information accuracy; 19‐item 5‐point Likert survey	Favorable overall: chatbot group showed greater timeliness, interaction, receptiveness and satisfaction, with reduced anxiety and perceived faculty workload; concern about incorrect information was also greater in the chatbot group	Short interaction period; outcomes based mainly on perceptions rather than objective learning; single institution; clinical context limited to a predoctoral implant clinic; potential novelty effect; concerns regarding accuracy and reliability
[12]	Huang et al. (2025)—Randomized student‐based educational intervention	4th‐ and 5th‐year undergraduate dental students at a Chinese university	187 analyzed (94 ChatGPT; 93 control)	ChatGPT used in addition to instructional videos for operative skills acquisition	Video‐based learning without ChatGPT	Skill‐performance score assessed using desktop virtual reality; cognitive load via pupil‐diameter changes; spatial ability; self‐efficacy; learning motivation	Favorable: ChatGPT group achieved higher skill‐performance scores (73.12 ± 10.06 vs. 65.54 ± 12.48; *p* < 0.001) and higher self‐efficacy (*p* = 0.04) and learning motivation (*p* = 0.02)	Short 1‐week intervention; single university; relatively short‐term outcomes; 5 students excluded after eye‐tracking calibration; effects on long‐term retention and transfer to clinical practice were not established
[13]	Rampf et al. (2024)—Randomized student‐based radiographic diagnostic training study	4th‐year dental students	55 analyzed; elaborated feedback *n* = 28, knowledge‐of‐results *n* = 27	Students worked with 16 virtual radiological cases for 8 weeks; AI system (dentalXrai Pro 3.0) was additionally evaluated for radiographic diagnostic performance	Elaborated feedback (eF) vs. knowledge‐of‐results (KOR) feedback for student learning	Detection of enamel/dentin caries and apical periodontitis; diagnostic accuracy, sensitivity, specificity, ROC‐AUC, image‐quality assessment; separate AI diagnostic performance	Mixed but generally favorable for elaborated feedback: eF improved enamel‐caries sensitivity (*p* = 0.037) and AUC (*p* = 0.020), and apical‐periodontitis accuracy (*p* = 0.011), sensitivity (*p* = 0.003) and AUC (*p* = 0.001). AI independently demonstrated high caries diagnostic performance (overall accuracy 0.976, AUC 0.971)	Only 55 complete datasets; 8‐week intervention; some outcomes showed no significant between‐group differences; AI was evaluated separately rather than directly assigned as the student feedback intervention; limited range of radiographic conditions; no assessment of treatment decisions or long‐term retention
[14]	Yilmaz et al. (2025)—Randomized controlled personalized‐feedback study	5th‐year dental students at Afyonkarahisar Health Sciences University	110; experimental *n* = 55, control *n* = 55	ChatGPT‐4o generated MeSH‐based personalized learning guides addressing individual learning gaps after incorrect diagnostic responses	Traditional correct/incorrect feedback without content‐specific guidance	15‐question radiographic diagnostic pre‐/post‐test; diagnostic performance; feedback clarity, usefulness, motivation and satisfaction	Strongly favorable: performance improvement was 3.6 ± 1.0 in the ChatGPT‐4o group vs. 1.3 ± 1.2 in controls (*p* < 0.001); final scores also favored ChatGPT (*p* < 0.001); clarity, usefulness, motivation and satisfaction were all significantly higher (*p* < 0.001)	Single institution; convenience recruitment; fifth‐year students only; no blinding; short educational follow‐up (1 month); ChatGPT‐generated outputs required expert review; applicability to other student levels and real clinical practice remains uncertain

Overall, the included studies report positive effects of educational interventions supported by AI on diagnostic reasoning, clinical decision‐making, learning performance, and student engagement. In addition, Fang et al. [11] reported that chatbot‐assisted teaching could be of great assistance for clinical teaching. Yet, the significant heterogeneity of interventions, educational contexts, study designs, and outcome measures made it difficult for us to pool the results numerically. Out of six studies, five basically were quantitative and onea mixed‐method exploratory study. The six studies were different in their educational research domains such as simulation‐based diagnostic training [9], classroom‐based virtual‐patient approach [10], medical learning with chatbot assistance [11], randomized ChatGPT‐assisted skills education [12], AI‐assisted radiographic diagnostic training [12], and personalized AI‐assisted feedback for radiographic diagnosis [14]. By categorizing the studies in this way, we tried to clarify the purpose of the educational intervention and the type of design method. Suárez et al. [9] and Jones et al. [10] predominantly assessed satisfaction, perceptions, engagement, and usability; Fang assessed mainly perceptions/interactions/acceptability of using medical chatbots. Stronger objective performance evidence came from the studies of Huang et al. [12] and Gokkurt Yilmaz et al. [14]. Rampf et al. [13] were able to assess both student outcomes and the performance of AI separately.

Risk of bias in included papers was evaluated using appraisal tools specific to the design: cross‐sectional study was assessed using the JBI Critical Appraisal checklist; pilot/implementation study was evaluated by the Mixed Methods Appraisal Tool (MMAT), whereas for the randomized studies, RoB 2 tool was used. In general, the studies showed a moderate to low risk of bias. The main drawbacks were non‐blinded participants, subjective evaluations of outcome, small or single‐center samples, and non‐control of confounding factors. None of the studies demonstrated an overall high risk of bias (Tables 4–6).

**Table 4:** Risk of bias assessment of the cross‐sectional study using Joanna Briggs Institute (JBI) Critical Appraisal Checklist: Low risk Some concerns High risk.

Domain	Suárez et al. [9]
1. Inclusion criteria clearly defined	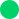 Yes
2. Study subjects and setting described in detail	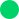 Yes
3. Exposure/intervention measured validly and reliably	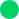 Yes
4. Objective/standard criteria for measurement	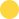 Unclear
5. Confounding factors identified	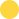 Unclear
6. Strategies to deal with confounding factors	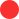 No
7. Outcomes measured validly and reliably	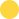 Some concerns
8. Appropriate statistical analysis	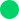 Yes
Overall risk of bias	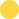 Some concerns

**Table 5:** Risk of bias/appraisal of the pilot implementation study using the Mixed Methods Appraisal Tool (MMAT): Low risk Some concerns High risk.

Domain	Jones et al. [10]
1. Appropriate sampling strategy	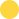 Some concerns
2. Sample representative/relevant to research question	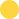 Some concerns
3. Appropriate measurement of outcomes	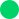 Yes
4. Complete outcome data	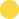 Some concerns
5. Appropriate analysis	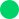 Yes
6. Risk of non‐response bias adequately addressed	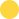 Unclear
7. Risk of researcher/observer bias	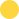 Some concerns
Overall risk of bias	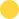 Some concerns

**Table 6:** Risk of bias assessment of randomized studies using the Cochrane Risk of Bias 2 (RoB 2) Tool: Low risk Some concerns High risk.

RoB 2 domain	Fang et al. [11]	Huang et al. [12]	Rampf et al. [13]	Yilmaz et al. [14]
D1. Bias arising from randomization	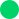 Low	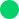 Low	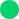 Low	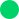 Low
D2. Bias due to deviations from intended interventions	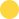 Some concerns	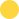 Some concerns	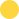 Some concerns	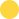 Some concerns
D3. Bias due to missing outcome data	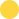 Some concerns	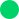 Low	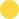 Some concerns	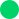 Low
D4. Bias in measurement of outcome	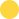 Some concerns	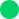 Low	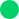 Low	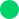 Low
D5. Bias in selection of reported result	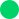 Low	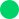 Low	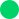 Low	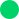 Low
Overall risk of bias	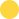 Some concerns	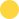 Some concerns	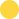 Some concerns	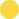 Some concerns

The study by Suárez et al. [9] demonstrated some concerns regarding the risk of bias. The study had clearly defined participants and a well‐described educational setting, and the chatbot intervention was consistently delivered. However, the study relied substantially on student‐reported outcomes and did not use randomization or a conventional control group, limiting the causal inference. Potential differences in students’ previous experience with technology and AI were also not fully controlled. Therefore, the study was considered to have some concerns overall, particularly regarding confounding and outcome measurement (Table 4).

The research of Jones et al. [10] has some degree of risk of bias, which is mainly because of its nature as an educational study exploring a novel method rather than a randomized control trial. In reality, the students were mainly recruited on a convenience basis, and the study was conducted in only one school, which could hinder the ability to extrapolate the results to other settings. Also, neither participants nor investigators were blinded to chatbot intervention, and several outcome measures reflected the aspect of usability, engagement, and learner experience. Despite such limitations, the intervention and classroom procedures were clearly stated, and the analysis was suitable for the exploratory nature of the study. Because of this, the overall assessment was classified as having some concerns (Table 5).

There were some concerns with the study reported by Fang et al. [11] as despite randomized allocation which would limit baseline selection bias, the participants could not be blinded to whether support was provided by a chatbot or conventional method. Several outcomes were based on participant perceptions and satisfaction, including their self‐assessment of performance, resulting in the risk of measurement and response bias. The small sample size and educational setting further limit the wider applicability of the findings. As a result, this was classed as having some concerns for the risk of bias, not a low risk in all areas. The study by Huang et al. [12] was again categorized as having some concerns overall on RoB 2. This was not only due to the inability to blind participants to the ChatGPT‐based educational intervention but also due to the use of a small number of courses for the educational setting. Randomization and use of an objective assessment of student performance mean that there was a low overall risk under the randomization and outcome‐measurement domains. There was some potential for bias from deviations from the intended intervention (ChatGPT‐based support) due to participants not being blinded, but as the intervention was educational in nature, and the participants knew their treatment group, some potential influence of motivation on performance or learning cannot be excluded, and so the study is overall assessed to have some concerns. The study by Rampf et al. [13] was assessed as having some concerns overall, despite the random allocation and objective criterion for diagnostic performance, as the participants could not be blinded to the feedback type and there was a small loss to post‐intervention data for four of the participants. The randomized comparison was between different feedback approaches, while the AI application was incorporated as part of the diagnostic‐learning environment; hence, the results should not be interpreted simply as an AI‐versus‐no‐AI comparison. Finally, the study by Yilmaz et al. [14] was scored as having some concerns overall as participants could not be blinded to the type of feedback in the study, and it was a single‐center study of fairly standard educational support. While the use of random allocation and the objective assessment of the radiographic diagnostic performance does reduce the potential bias, there is a potential for bias in motivation due to the nature of the experimental group receiving tailored ChatGPT feedback, resulting in overall some concerns (Table 6).

### 3.1. Overall Interpretation

All studies were assessed as carrying a low overall risk of bias. Randomization provided good protection against selection bias, but the main problem with all studies was the inability to blind participants to AI‐based educational interventions, which may have caused biased assessment of outcomes. Uncertainty was further increased by subjective measurements, limited participant numbers, single institutional settings, and little control of possible confounding. So, the results indicate that AI‐powered chatbots have educational value, but these results are preliminary given the methodological heterogeneity and the existing risk of bias.

Quantitative synthesis and publication bias evaluation could not be carried out since the six studies diverged in research methods, AI interventions, comparators, outcome measures, and statistical reporting. In addition, formal publication bias assessment methods like funnel plots and Egger’s test are regarded to be unreliable in particular when the studies are not homogeneous. Hence, the findings were reviewed narratively.

## 4. Discussion

### 4.1. Summary of Main Findings

We evaluated AI‐driven chatbots and LLMs in university‐level dental education with respect to their educational applications, effectiveness, usability, and the considerations around their implementation. We included six studies in our final synthesis [9–14]. The included studies described different educational applications, such as diagnostic reasoning, classroom‐based chatbot implementations, AI‐based assistance with clinical teaching, instruction of skills using AI, radiographic diagnostic support, and the generation of AI‐based feedback. Given the significant heterogeneity in the different study designs, tested interventions and their comparators, evaluation metrics, and outcome reporting, we opted for a narrative synthesis rather than a quantitative synthesis.

Based on the studies included, AI‐based interventions may have a positive impact on specific objective educational outcomes such as diagnostic performance, the acquisition of clinical skills, and the interpretation of radiographs [9, 12–14]. Positive effects on subjective outcomes, such as engagement, satisfaction, perceived usefulness, and the learning experience, have been described [10, 11]. However, AI‐based chatbots do not automatically improve dental education, and positive impacts on educational outcomes should not be assumed. The current evidence base remains small and methodologically heterogeneous, and the answers to important questions, such as the accuracy and boundaries of AI, the appropriate in‐class integration, and their ethical use, remain largely unanswered.

The broader literature deals with the transformative potential of AI for dental education while recognizing the importance of human oversight and critical reflection [1–8]. These background articles provide the conceptual background for the findings presented here but were not included in the six evidence synthesis studies.

### 4.2. Objective Educational Outcomes

Objective educational outcomes yielded the strongest evidence in the current review. Suárez et al. [9] studied an AI‐based chatbot that simulates virtual patients and enables repeat presentation of diagnostic cases and the development of diagnostic reasoning for dental students. Their study shows that conversational AI can potentially support students by exposing them to clinical scenarios in a planned learning sequence. This is important because virtual patients can provide diagnostic reasoning practice, even without a real patient, and help students prepare for more complicated clinical situations.

Huang et al. [12] published a randomized study, which was the first to evaluate ChatGPT as an instructor in dental skill education. In this study, 187 dental students were assigned to the group that used ChatGPT or to the group that used video‐based learning as a control. Later, skill performance was assessed using a desktop VR system, and it was determined that the ChatGPT group performed better. Therefore, this study shows that AI systems, in this case, ChatGPT, can be used as a complement to traditional education systems beyond just being an information lookup tool.

Rampf et al. [13] studied the radiographic diagnostic competence of fourth‐year dental students using different formats of feedback and the incorporation of AI systems. The study used diagnostic outcome measures of accuracy, sensitivity, specificity, and area under the curve. The authors concluded that feedback was an important factor for diagnostic learning, while the AI component was usable within the educational context. The authors want to make it clear that the study is not an AI versus no AI randomized control trial because the randomized control group was primarily focused on different feedback. The group was then placed in different learning environments, which included an AI system.

Yilmaz et al. [14] studied the effect of ChatGPT‐generated AI‐assisted personalized feedback and conventional correct/incorrect feedback on radiographic diagnostic performance of dental students. Personalized feedback based on AI may help improve the radiographic diagnostic performance. This study helps differentiate providing students with information generated by AI and using AI to develop individualized feedback based on the performance of the learner.

When the studies are considered as a whole, it shows that the most promising objective applications noted in this review are active learning, clinical reasoning, simulation, diagnostic interpretation, and personalized feedback, not passive information delivery [9, 12–14]. However, because there are only a few studies and the educational tasks vary, it is not possible to establish whether one particular AI method is consistently better than that of traditional teaching.

### 4.3. Subjective Outcomes, Learner Perception, and Usability

The studies mentioned also offer evidence relating to the students’ experience and the usability of the systems. Jones et al. [10] assessed the way in which AI chatbots were used in the classroom, these chatbots being designed to simulate an interaction between a dental patient and a clinical supervisor. The study focused on student engagement and usability, showing that conversational AI has the potential to produce interactive experiences in the classroom. The results support the idea that AI role‐play can give students the opportunity to engage in repeated clinical communication and reflection within a controlled educational setting. Fang et al. [11] looked at a specially designed chatbot intervention in the context of clinical dental education and compared it with a conventional online learning platform. Their results showed that students had favorable views of chatbot‐assisted learning, covering areas such as the quality of interaction, the timeliness of responses, the chatbot’s receptiveness, and overall satisfaction. At the same time, however, the students raised worries about the possibility of the chatbot producing inaccurate information. This combination of high usability together with concerns about reliability is especially significant for dental education since the fact that something is perceived as useful does not mean that it is educationally accurate. The subjective findings thus add to the objective evidence. Although AI can make learning more interactive, accessible, and engaging, it is wrong to regard positive student opinions as equivalent to actual improvements in competence. This point is particularly important since satisfaction and engagement can be affected by the novelty of generative AI and by students’ expectations regarding the technology.

The existing literature has also found both enthusiasm and uncertainty about the use of generative AI in dental education. Among the reviews and conceptual papers, there has been a recognition of the potential for personalization and interactive learning, together with concern about misinformation, academic integrity, the development of critical thinking, and over‐reliance on AI‐generated content [3, 6–8, 15–17].

### 4.4. Implementation and Educational Integration

The six studies shown illustrate the various ways in which AI can be introduced into the dental education. Suárez et al. [9] applied a virtual‐patient model in order to aid diagnostic reasoning, Jones et al. [10] carried out the use of a chatbot in role‐play as part of classroom teaching, Fang et al. [11] looked at the use of chatbots in clinical learning, Huang et al. [12] incorporated ChatGPT into skills education, and Rampf et al. [13] together with Yilmaz et al. [14] assessed AI‐assisted feedback in the area of radiographic diagnosis. This version indicates that the educational value of AI will to a large extent depend on the way in which the technology is incorporated into the curriculum and not merely on the fact that AI is available. It seems especially appropriate for tasks that involve repeated interaction, immediate feedback, scenario‐based reasoning, and personalized learning support. Nevertheless, such applications must remain linked to clearly established learning objectives and faculty‐supervised educational activities. It can therefore be regarded as useful that AI chatbots should mainly serve as educational aids rather than as substitutes for teachers or traditional clinical teaching. The teacher must still be responsible for setting the competencies, verifying the learning material, detecting errors, overseeing clinical reasoning, and making sure that the students acquire the ability to form their own professional judgments. This view is in agreement with the wider existing literature, which stresses the importance of maintaining critical thinking and human supervision when AI is incorporated [3, 7, 8]. Accuracy, ethical considerations, and responsible use accuracy and reliability are important factors that have come out of the studies cited. Fang et al. [11] did express concerns about the incorrect information produced by the chatbot even though they had a favorable view of its educational value. This result is especially significant in the context of dental education since students might then apply the information they have learned to patient care. The possibility of AI‐generated information being inaccurate or made up—something commonly referred to as hallucination—means that there is a need for a systematic check of educational material derived from AI. Just as background studies and reviews have highlighted concerns about accuracy, bias, academic misconduct, privacy, and excessive reliance on generative AI [1, 5–8, 11, 17]. The use of AI also brings up issues concerning academic integrity and the extent to which students become dependent. Should students mainly use AI in order to get answers rather than to enhance their abilities in reasoning, reflection, and problem‐solving, the technology might instead weaken rather than improve the aims of education. For this reason, dental curricula ought to stress AI literacy, include source verification, require the disclosure of AI usage in appropriate cases, and involve a critical assessment of the responses produced by AI. When it comes to ethical implementation, privacy and data governance should also be taken into account—especially in cases where information about patients or clinical scenarios is entered into external AI systems. Before widespread implementation, faculty members should set up clear institutional policies concerning the use of AI, data protection, attribution, assessment, and academic integrity [18]. The methodological limitations of the included studies. The methodological quality of the six studies which were included showed variation. The evidence comprised exploratory, implementation, comparative, and randomized designs rather than a single group of controlled trials [9–14]. This diversity prevents a direct comparison of their educational effects. A number of methodological issues were spotted. First, it was generally either difficult or impossible to blind the participants since the students knew when they were dealing with an AI‐assisted intervention. This could have had an effect on their motivation, level of engagement, and the outcomes they reported. Second, in some of the studies, partial reliance was placed on subjective measures such as satisfaction, usability, perceived usefulness, or the learners’ experience [10, 11]. Even though these outcomes are relevant from an educational point of view, they are more prone to response and expectation bias than objective evaluations. Third, several of the studies were carried out within individual institutions or with rather narrow groups of students, which limits the extent to which the results can be applied to other dental schools, educational systems, countries, and levels of training. Fourth, the sample sizes were relatively small in some of the studies, thereby decreasing the statistical precision. Fifth, the interventions varied greatly in terms of the type of AI, the educational task, the length of exposure, the feedback mechanism, and the comparator. The randomized studies offered more convincing evidence than the exploratory ones, but even those still need to be interpreted with caution. Huang et al. [12] employed randomization together with an objective evaluation of skill, whereas Rampf et al. [13] used randomly allocated feedback and objective radiographic results. Yilmaz et al. [14] likewise used a controlled approach with objective diagnostic outcomes. However, since the participants were not blinded and the studies were carried out in an educational context, there are potential sources of bias. As a result, the overall assessment of the risk of bias showed some concerns rather than indicating a uniformly low risk in all studies.

### 4.5. Limitations of the Review Process

This review also has some limitations: (a) only six studies met the inclusion criteria; a relatively small number of studies. While the search methods were extensive by including four bibliographic databases and screening of reference lists from included reports, relevant studies could have been missed due to the dynamic nature of the vocabulary used to describe generative AI chatbots, virtual patients, and educational applications. (b) Only English publications were included. (c) A formal quantitative analysis was not performed because the six included studies varied too much in terms of study design, interventions, comparators, outcome measures, and statistical results. In addition, a formal assessment of publication bias was not performed since only six reports were included and the analyses would not be informative given the lack of homogeneity between the included studies. (d) Several relevant reports on AI in dentistry were excluded from consideration because they did not meet the eligibility criteria of educational interventions. While these reports might contain pertinent information, we choose to exclude them in order to maintain an analytical focus on dental education at the university level.

#### 4.5.1. Implications for Dental Education

Based on the findings, it could be posited that AI chatbots have the most value as an educational tool when used as an addition to standard curricula, for training virtual patients, diagnostic or other skills, radiographic or general diagnosis, and feedback. They should be used as a supplement to standard faculty‐supervised learning rather than a substitute.

A blended approach where faculty sets the learning objectives, establishes competencies, and provides overarching instructions while students use AI to practice and receive feedback in an individualized manner and as an extension of standard learning appears most reasonable. This would allow for increased practice opportunities while maintaining the supervisory role of faculty. At the same time, faculty needs to set up the expectations for using such tools, and students need instruction on how best to utilize these opportunities within the framework of the overarching educational goals. Both students and faculty need AI literacy to utilize such tools effectively, including understanding when the information provided by AI is incorrect or inadequate, accessing information from other sources, and critically evaluating the information provided by AI, while maintaining the faculty’s supervisory role and avoiding potential academic misconduct. AI‐driven chatbots have also shown promising results in delivering patient‐friendly and accurate information on dental implants [19], dental prosthesis [20], and promoting oral health in young children [21, 22]. Studies have also shown mixed results when using Ai‐derived chatbots for the purpose of conducting examinations [23, 24].

#### 4.5.2. Implications for Future Research

The future research is needed to go beyond proof‐of‐concept and small single‐center studies. Larger multi‐center RCTs with sufficient power to demonstrate educational efficacy are needed. The choice of outcome measures needs to be standardized and validated across institutions to allow for comparison of results between these studies and enable future meta‐analyses of the available evidence. Future studies should report detailed information on participants, interventions, comparators, adherence, and outcome measures to allow for replication of effective interventions. Particular attention should be paid to describing the characteristics of the included populations, intervention components (including duration), prompts used, specific version/model of the AI, specific details of the use of AI (e.g., specific prompts used, if any), comparator intervention, adherence to the intervention, and outcome measures and their timing.

It is also essential that future studies include objective outcome measures, including objective measures of diagnostic accuracy, reasoning, and other competencies, along with measures of satisfaction. Additionally, future studies should assess long‐term learning and retention of newly acquired competencies following the implementation of the AI‐assisted educational interventions. Comparative studies are needed to assess the relative efficacy of the different types of AI‐assisted interventions, including chatbots, virtual patients, and other methods of instruction or feedback, to determine which has the most value for postgraduate dental education. Future studies also need to assess whether AI‐assisted educational interventions lead to improved performance and do not undermine critical reasoning and appropriate use of AI.

Furthermore, future studies need to address the issues of AI accuracy, ethical issues associated with its use, and privacy and confidentiality concerns of students and patients. Finally, future studies need to explore and address the framework for faculty supervision, including determining when such supervision is needed, how it could be structured, and whether faculty involvement affects student performance.

#### 4.5.3. Overall Interpretation

In summary, the current review finds that there is preliminary evidence that AI chatbots and LLM‐assisted educational interventions can be of benefit within university‐level dental education. The strongest evidence comes from objective assessments of specific skills, namely, reasoning, with several studies demonstrating that AI‐assisted practice leads to improvement on objective measures such as diagnostic accuracy, skill performance, and radiographic diagnosis [9, 24,27,28], while the implementation studies highlight the advantage of such methods in terms of engagement, ease of use, and satisfaction [10, 14]. The overall applicability of these findings is limited by the heterogeneity of the interventions, sample sizes, the lack of blinding, reliance on self‐reported outcomes, and single‐center nature of most of the included studies. It is therefore too early to conclude that faculty education can be substituted by AI‐assisted methods. Rather, it appears that AI‐assisted practice should be used as an addition to standard curricula with faculty instruction remaining integral to postgraduate dental education. Future research should focus on identifying effective AI‐assisted interventions, including those that utilize LLMs, as well as long‐term educational outcomes following their implementation to guide incorporation into dental education.

The comparison suggests that there is value in both approaches to developing AI‐assisted educational technologies for postgraduate dentistry. Rule‐based chatbots appear to have advantages in terms of addressing standard competencies, particularly with respect to standardized protocols for procedures or patient management, while LLMs have greater potential in terms of individualized instruction, diagnosis, and reasoning. At the same time, the flexibility and increased complexity of the LLMs appear to come at the cost of decreased reliability, ethical concerns, potential breaches of privacy and academic integrity, and reduced faculty oversight. Thus, the evidence provided by this review suggests that a blended approach to AI‐assisted postgraduate dentistry education is most appropriate, with both types of interventions finding their use depending on the competency targets and with faculty oversight being maintained. This view is compatible with the current state of evidence, though more well‐designed RCTs, including studies comparing different approaches, are needed to confirm these findings. This viewpoint also incorporates the considerations of ethical concerns, faculty supervision, and potential academic misconduct related to the use of AI in postgraduate dental education (Table 7).

**Table 7:** Comparative educational characteristics and proposed integration of AI chatbots and large language models in university‐level dental education.

Parameter	AI chatbots (rule‐based/task‐specific)	Large language models (LLMs; e.g., GPT‐based)	Implications for dental education
Core functionality	Predefined responses, structured workflows, task–oriented interaction	Context‐aware, generative, adaptive conversational interaction	Select the technology according to the educational task and required level of adaptability
Educational applications	Virtual‐patient simulations, procedural training, behavioral guidance	Clinical reasoning, interactive tutoring, academic support, feedback and communication training	Use structured chatbots for protocol‐driven learning and LLMs for complex, interactive learning
Personalization	Limited and dependent on programmed pathways	Greater personalization through dynamic, context‐sensitive responses	LLMs may support adaptive learning pathways when appropriately supervised
Clinical reasoning support	Moderate; primarily algorithm‐ or decision‐tree based	Potentially greater support for multistep reasoning and differential‐diagnosis exercises	LLM‐based reasoning activities should be validated and faculty supervised
Accuracy and reliability	More consistent within predefined tasks	Variable; potential for hallucination, misinformation and inconsistent responses	Human verification and validated educational content are essential, particularly for clinical applications
Student engagement	Moderate; predictable interaction	Potentially high through conversational and interactive learning	Interactive AI may support self‐directed and active learning
Assessment utility	Suitable for standardized, structured formative exercises and simulations	Can generate questions, explanations and individualized formative feedback	Structured chatbots may support standardization, whereas LLMs may complement formative feedback
Scalability	Generally easy to deploy for defined tasks	Highly scalable but may require greater computational and governance resources	Institutional infrastructure, cost and technical capacity should guide implementation
Ethical and academic concerns	Relatively limited when outputs are tightly controlled	Greater concerns regarding hallucination, bias, privacy, academic misuse, and over‐reliance	AI literacy, ethical guidance, privacy safeguards and faculty oversight are required
Implementation complexity	Low to moderate	Moderate to high because of monitoring, validation and updating requirements	A phased implementation strategy is preferable
Evidence from the present review	Evidence includes structured simulation and chatbot‐based educational applications	Evidence is emerging, including LLM‐supported instruction and personalized feedback	Current evidence supports cautious educational integration; additional rigorous comparative trials are required
Most appropriate educational role	Protocol‐driven learning, simulation, behavioral and skill‐based training	Adaptive tutoring, clinical reasoning, communication and individualized feedback	A complementary or hybrid model, rather than replacement of conventional teaching, is recommended

## 5. Conclusion

AI‐driven chatbots and LLMs show potential as supportive tools in university‐level dental education, particularly for learning, clinical reasoning, simulation, and individualized feedback. However, the available evidence is limited and heterogeneous, with concerns regarding accuracy, ethics, and appropriate use. AI should therefore complement, rather than replace, conventional dental teaching, with further rigorous research needed to establish its educational effectiveness and safe implementation.

## Author Contributions

**Ramnarayan B. K.**: conceptualization, data curation, drafting manuscript, critical revision of manuscript, admin, technical or material support, supervision, final approval. **Raghad Hashim**: conceptualization, formal analysis, critical revision of manuscript, statistical analysis, final approval. **Krishnanand P. S.**: conceptualization, data curation, critical revision of manuscript, admin, technical or material support, supervision, final approval. **Vidya M. A.**: conceptualization, data curation, drafting manuscript, final approval. **Alexander Maniangat Luke**: conceptualization, formal analysis, critical revision of manuscript, final approval. **Usha C.**: conceptualization, data curation, critical revision of manuscript, statistical analysis, final approval.

## Funding

This study was self‐funded.

## Ethics Statement

The Systematic Review has been registered with Open Science Framework Database DOI: 10.17605/OSF.IO/8AEVF.

## Conflicts of Interest

The authors declare no conflicts of interest.

## Data Availability

No new data has been generated for the study.
